# The Application of a SaCoVLM^TM^ Visual Intubation Laryngeal Mask for the Management of Difficult Airways in Morbidly Obese Patients: Case Report

**DOI:** 10.3389/fmed.2021.763103

**Published:** 2021-11-18

**Authors:** Yongtao Sun, Linlin Huang, Lingling Xu, Min Zhang, Yongle Guo, Yuelan Wang

**Affiliations:** ^1^Department of Anesthesiology, The First Affiliated Hospital of Shandong First Medical University & Shandong Provincial Qianfoshan Hospital, Shandong Institute of Anesthesia and Respiratory Critical Medicine, Jinan, China; ^2^Department of Anesthesiology, Shandong First Medical University, Jinan, China

**Keywords:** SaCoVLM^TM^, visual intubation laryngeal mask, difficult airways, morbidly obese patients, laryngeal mask airway

## Abstract

We report insertion of the SaCoVLM^TM^ in three awake morbidly obese patients (BMI 46. 7–52.1 kg/m^2^). The patients were given intravenous atropine and midazolam injections after entering the operating room and then inhaled an anesthetic with 2% lidocaine atomization. After SaCoVLM^TM^ insertion while patients were awake, when the vocal cords were visualized, controlled anesthetic induction commenced with spontaneous ventilation. The entire anesthesia induction and intubation process was completed under visualization, and no adverse events such as hypoxemia occurred. No patient had an unpleasant recall of the procedure. We conclude that the SaCoVLM^TM^ is easy to use, well tolerated and suitable for awake orotracheal intubation in patients with known difficult airways.

## Introduction

**Figure 1:**
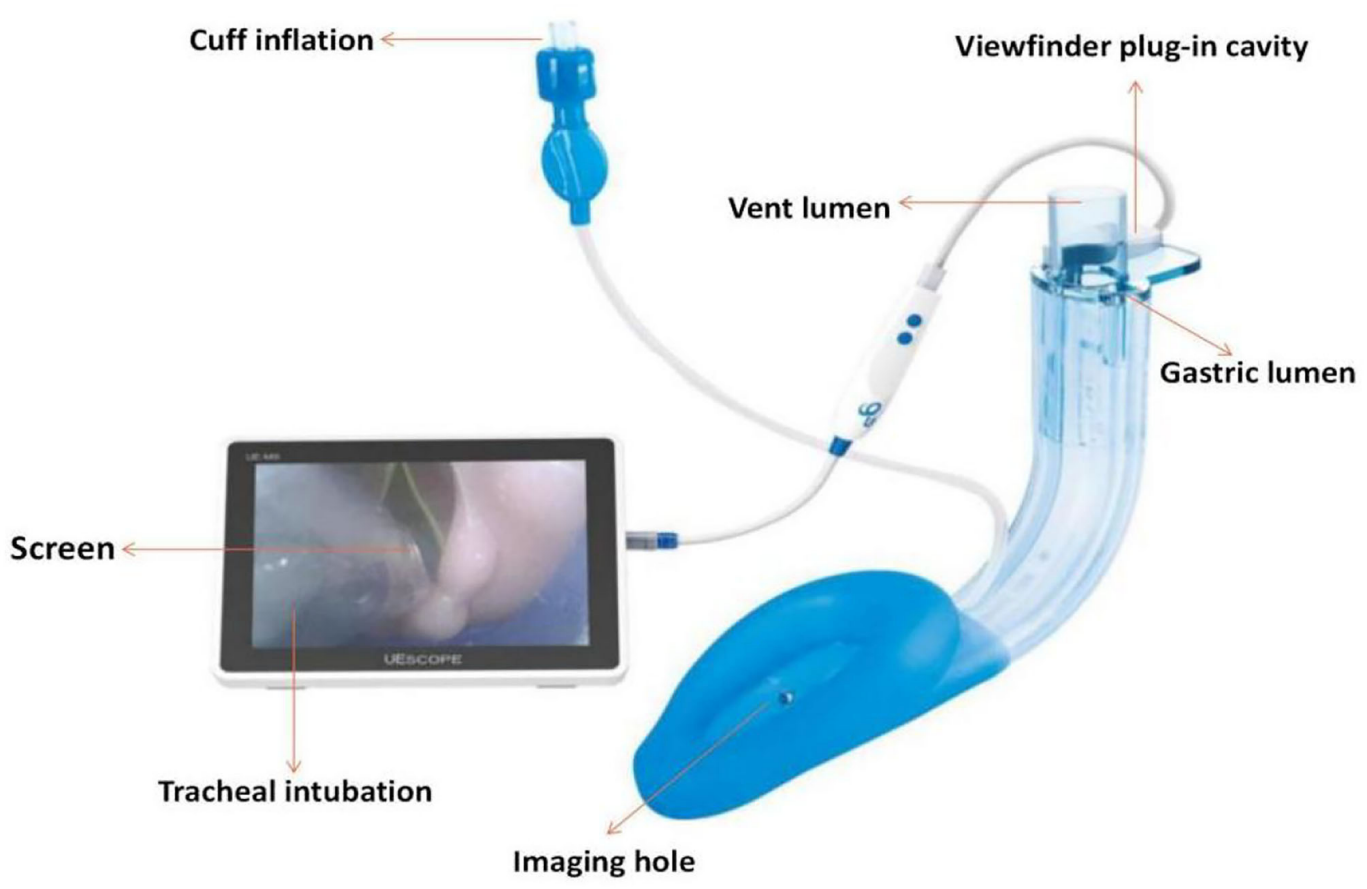
SaCoVLM^TM^ visible intubation laryngeal mask,photo courtesy of Youyi Medical Instrument Co. Ltd. (HangZhou, China).

Awake tracheal intubation is recommended in patients with known or predicted difficult airways (1). The most widely used technique for awake intubation is the fibreoptic bronchoscope (FOB). New optical intubation devices have been developed and are currently being used as an alternative method for this purpose, but only a few cases of awake intubation have been reported (2–4). The SaCoVLM^TM^ visible intubation laryngeal mask was independently developed in China. It is a three-cavity laryngeal mask that provides ventilation with a visual interpolation cavity and gastric duct cavity (Figure 1). Through the visual appliance external display screen, the laryngeal housing and tracheal intubation can be visualized, and the position of the laryngeal mask can be continuously monitored during the perioperative period. The intubating laryngeal mask airway (ILMA) is a supraglottic airway that facilitates ventilation and blind tracheal intubation (5). When using the ILMA, however, anesthesiologists must blindly try to optimize the position of the throat mask to meet the appropriate intubation conditions. Using SaCoVLM^TM^, we can directly observe the impact of standard reset operations (e.g., Chandy and “UP-Down” operations (6, 7)) and visually insert the tracheal intubation device. We report the first case series utilizing the SaCoVLM^TM^ LMA (Youyi Medical Instrument Co. Ltd., HangZhou, China) as an awake tracheal intubating device in patients with an anticipated difficult airway management.

## Case Presentation

With Institutional Review Board approval and written informed consent from all patients, we collected SaCoVLM^TM^ insertion data on three morbidly obese patients who then received robot-assisted laparoscopic sleeve gastrectomies under general anesthesia. All three patients denied any history of gastric reflux, and all had been fasting for a minimum of 10 h.

### Patient One

A 38-years-old, 124 kg, 163 cm, BMI 46.7 kg/ m^2^, American Society of Anesthesiologists Classification (ASA) III level female. She had a history of 5 years hyperthyroidism (propylthiouracil 300 mg qd.) and Obstructive sleep apnea (OSA) for over 10 years, abdominal hernia for over 2 years, habitual mandibular dislocation, thyroidectomy and two cesarean deliveries without any significant personal or familial past history. Long-term high calorie diet and eating habits led her to gain weight gradually. Preoperative airway assessment included a Mallampati class III, a 7.5 cm thyromental distance, a 5 cm interincisor distance and a 46 cm neck circumference. Physical examination showed nutritional obesity, abdominal distension, two old transverse scars in the lower abdomen, and a transverse scar of about 5 cm in the neck. Admission diagnosis: Morbidly obese, Obstructive sleep apnea, Abdominal hernia, Hyperthyroidism, After thyroidectomy, After cesarean section.

### Patient Two

A 48-years-old, 140 kg, 164 cm, BMI 52.1 kg/m^2^, ASA III female. She was hospitalized for symptomatic angina. Owing to a gigantic appetite mainly on rice and pasta, her weight gradually increased in the past 20 years. Both her parents and sibling 3 were over obese. She had a history of hypertension (Bisoprolol 5 mg, Amlodipine 10 mg, and Olmesartan 40 mg), OSA, Diabetes, fatty liver and umbilical hernia, aortic stenosis and hypercholesterolemia. Preoperative airway assessment included a Mallampati class III, a 7.0 cm thyromental distance, a 5 cm interincisor distance and a 41 cm neck circumference. Physical examination showed nutritional obesity and mild cyanosis of the lips. Hematologic tests shows: WBC 10.93 × 10^9^ /L, NEU% 0.798, CRP 11.7 mg/mL. CT showed fatty liver, umbilical hernia and dilatation of surrounding intestine. Admission diagnosis: Morbid obesity, Umbilical hernia, Hypertension, Fatty liver.

### Patient Three

A 29-years-old, 145 kg, 172 cm, BMI 49.0 kg/m^2^, ASA III male. His past medical history include liposuction surgery 3 years ago, hypertension, OSA and diabetes. progressive weight gain over 20 years, sleep snoring and gasping suppression for 5 years. By restricted diet, exercises he didn't get a good weight control and at home for Intermittently ventilator assisted breathing therapy at present. Preoperative airway assessment included a Mallampati class III, a 7.0 cm thyromental distance, a 5 cm interincisor distance and a 45 cm neck circumference. Physical examination showed nutritional obesity, abdominal distension, generalized fat accumulation. Hematologic tests shows: AST 52.10 U/L, UA 559.0 μmol/L, INS μIU/mL, UCP 5.25 ng/mL, SpO_2_70.00 mmHg, GLU 6.40 mmol/L. Admission diagnosis: Morbid obesity, Obstructive sleep apnea.

All three patients were managed using the same protocol. The patients underwent routine monitoring and were given 2 mg of midazolam and 0.4 mg of atropine intravenously after being transferred to the operating room (OR). Aerosolized inhalation of 2% lidocaine was performed in the semisitting position on the transfer bed for 15–20 min without subglottic anesthesia. Each patient was placed in a slope position, the radial artery was punctured and catheterization under local anesthesia was performed to monitor invasive arterial blood pressure, and an intravenous micropump infusion of dexmedetomidine was given (load 1 μg·kg^−1^·10 min^−1^, maintenance amount 0.5 μg·kg^−1^·h^−1^ until 40 min before the end of the operation).

**Figure 2:**
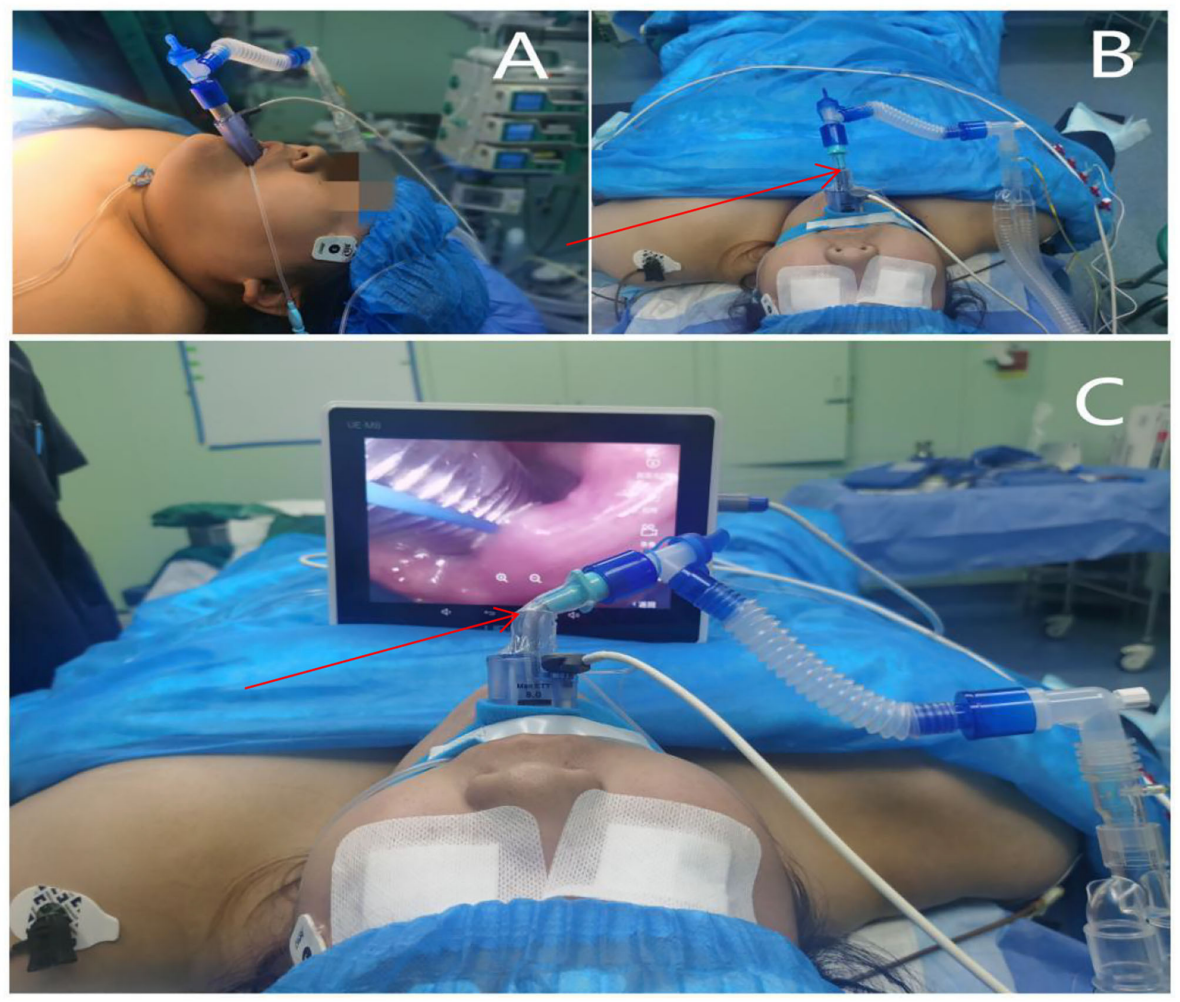
Airway management, Fixed place of infusion paster (Ventilation lumen entrance and tracheal intubation).

A laryngeal tube was used to test the sensation in the back of the oropharynx. If there was a pharyngeal reflex, 2 mL of 2% lidocaine was sprayed. The laryngeal mask model was chosen according to the patient's lean body mass. The SaCoVLM^TM^ No. 4 was used for patients one and two and the SaCoVLM^TM^ No. 5 was used for patient three chooses. All patients tolerated the procedure well, were connected to the anesthesia circuit, and received 100% oxygen. The glottis was directly observed in 2 patients, and the epiglottis was folded down in 1 patient. Through the “UP-Down” operation (slowly withdrawing the cuff from the pharynx 5-6 cm to aid in unfurling the epiglottis, and then re-inserting), a glottis view was obtained (Figure 2A). Once we observed the glottis, we observed the waveform of end-breathing carbon dioxide.

**Figure 3:**
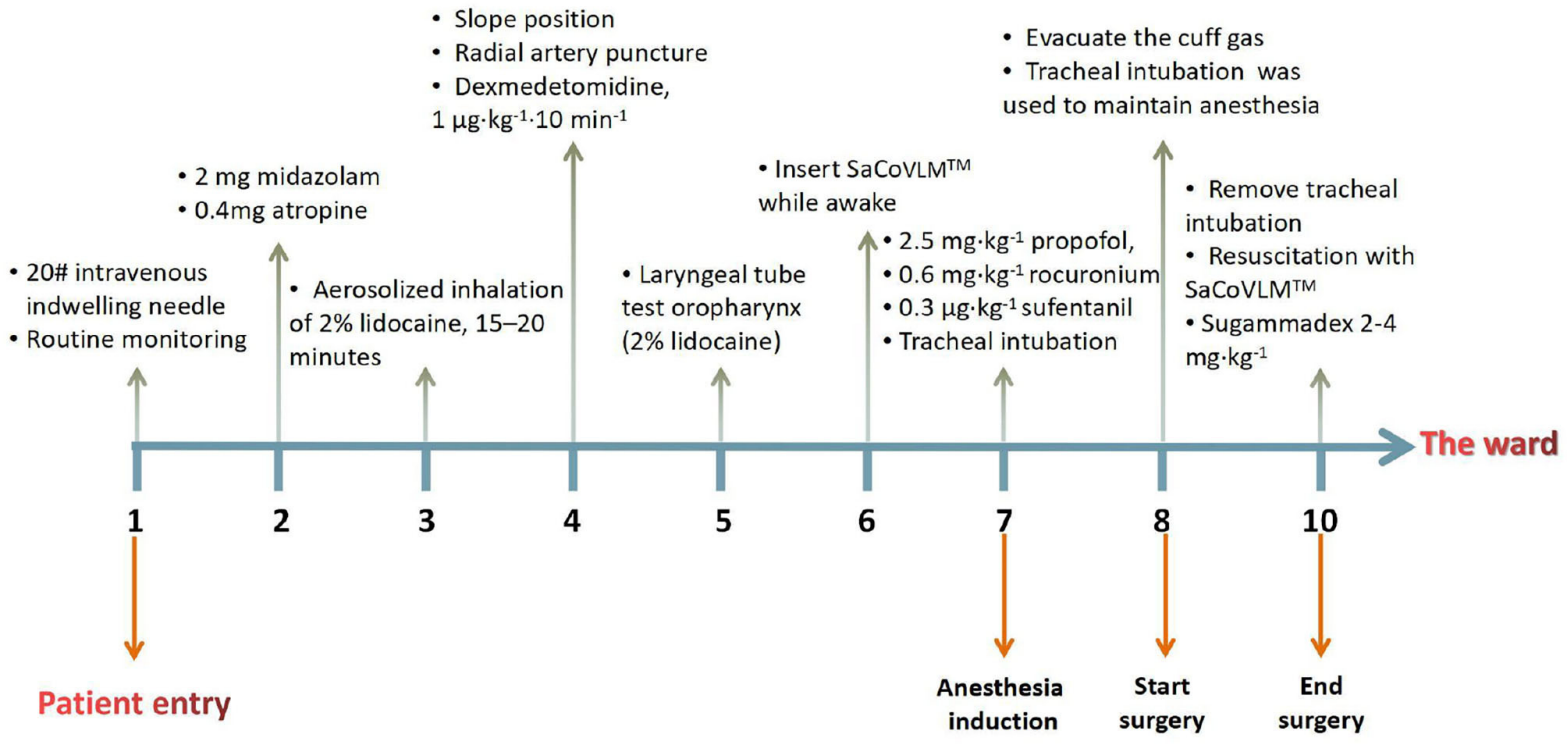
Anesthesia management timeline.

For the induction of anesthesia, 2.5 mg·kg^−1^ of propofol, 0.6 mg·kg^−1^ of rocuronium and 0.3 μg·kg^−1^ of sufentanil were immediately injected. When the BIS value reached 40–60, visual descending tracheal intubation was performed, and the 3 patients successfully completed tracheal intubation (Figure 2B). The tracheal intubation and the entrance of the LMA was fixed with the infusion paster, the laryngeal mask was kept to evacuate the cuff gas, and tracheal intubation was used to maintain anesthesia during the operation (Figure 2C). Forty milliliters of 0.375% ropivacaine was chosen, and bilateral transversus abdominis nerve blocks were conducted under ultrasound guidance. An intraoperative pump injection of propofol 4–12 mg·kg^−1^·h^−1^ and remifentanil 0.2–0.5μg·kg^−1^·min^−1^ was used to maintain anesthesia; after the operation, tracheal intubation was removed under deep anesthesia. The laryngeal mask was kept and transferred to the post-anesthesia care unit (PACU). During the resuscitation period, sugammadex sodium was used to antagonize muscle relaxation at a dose of 2–4 mg·kg^−1^. After the patients were awake, the laryngeal mask was well tolerated. After 1 h of observation, the laryngeal mask was removed. All 3 patients had no adverse reactions. They were safely transferred to the ward after continued observation for 1 h. The dosage of the above patients was calculated based on their lean body mass, the anesthesia management timeline are depicted on Figure 3.

On the first postoperative day, an interview was conducted with each patient on their induction and intubation experience. All 3 patients could recall being pushed to the operating room and had memories of procedures such as indwelling intravenous needles and aerosol inhalation. One patient had no memory of SaCoVLM^TM^ implantation, and two patients described it as if they had swallowed a very large object. After midazolam was given, all patients clearly denied fear or discomfort. All patients were willing to experience the same anesthesia again, and their satisfaction with anesthesia was high. The postoperative VAS scores were all below 3, and no complications, such as hypoxemia, nausea or vomiting, occurred.

## Discussion

This is the first reported series of awake insertion of the SaCoVLM^TM^ in morbidly obese patients. SaCoVLM^TM^ has shown advantages in clinical applications since its creation in 2018. Visualization of laryngeal mask placement and endotracheal intubation and Airway patency as well as 100% oxygen delivery was achieved. we can continuous monitoring the LMA location during Perioperative period. SaCoVLM^TM^ is comparable in price to a common LMA that can easily clinical promoted, nevertheless, it's easy to use, well tolerated and safe (with a 30 cm H_2_O expected seal pressure). However, SaCoVLM^TM^ has some limitations. Its imaging was not sufficiently sharp and the only way to improve imaging quality by adjusting the LMA position was not flexible enough. Supplementary intubation instruments such as fibrobronchoscopy and bougie are sometimes required. Susceptibility to airway secretions makes suppressive secreting drugs (e.g., atropine, pentylenetrin) a reliance which is limited to exceptional patients (Glaucoma patients). At present, clinical research to confirm the application of SaCoVLM^TM^ is still needed.

Studies have shown that ILMA can be successfully placed in conscious patients (5). Combes et al., for example, reported the effectiveness of ILMA in morbidly obese patients (8). LMA CTrach™ is the world's first visual intubation laryngeal mask and was applied in clinical practice in 2004. In 2006, Liu et al. (9) first reported the use of the LMA CTrach™ in 84 normal volunteers. The first intubation success rate was 100% when the glottis could be seen in the center of the monitor. A number of studies have shown that LMA CTrach™ has greater advantages in the treatment of difficult airways, including in morbidly obese patients, and for airway resuscitation, abnormal head and neck movements and other difficult airway treatment problems (10). In addition, LMA CTrach™ was not well clinically promoted due to its exorbitant price. The usage of the SaCoVLM^TM^ has overcome such difficulties. Although it does not provide a clear image like other fiber optic devices, the glottis can usually be seen and centered on the monitor, which is enough to successfully complete the first tracheal intubation.

Video-guided insertion can better locate the glottis, provide a more unobstructed airway, and improve the first success rate of tracheal intubation and gastric drainage tube insertion. Blind probe insertion into the supraglottic airway leads to poor positioning, downward folding of the epiglottis, and the use of more auxiliary devices, so the incidence of complications is higher (11). In fact, the Difficult Airway Society (DAS) guidelines state that “blind” airway management techniques are unreliable and are related to the high incidence of airway trauma (12, 13). Unlike traditional video laryngoscopes, the SaCoVLM^TM^ reduces the need for assistive devices. It can control the airway within a few seconds and establish the best ventilation for tracheal intubation, reducing the time of apnea, which is suitable for patients with poor physiological reserve. The SaCoVLM^TM^ is especially beneficial for certain patients such as morbidly obese patients or pregnant women.

The main cause of anesthesia-related injuries is respiratory safety, which cannot be ensured (12, 13). Actually, the most critical anesthetic skill for such patients is safe airway management, and a key component of this skill is the mastery of multiple techniques for securing the airway. When faced with a known or anticipated difficult-to-manage airway, although awake fiberoptic bronchoscopy may constitute “plan A” for intubation, a “plan B” is required, as fiberoptic bronchoscopy may fail to lead to successful tracheal intubation (e.g., due to airway secretions or blood). All our patients had both a high Mallampati score and a thick neck circumference, a combination that is known to be a strong predictor of difficult airway (14). The use of the SaCoVLM^TM^ helped achieve safe, successful intubation while maintaining spontaneous respiration. As the vocal cords were always kept in view, we did not need to be concerned about the displacement of laryngeal structures that may occur with the induction of anesthesia. Awake SaCoVLM^TM^ insertion requires only that patients have adequate topical anesthetic applied to the oropharynx, whereas awake tracheal intubation using fiberoptic bronchoscopy requires that the infraglottic structures be anesthetized as well. The issue of infraglottic anesthesia is important in considering a patient's ability to protect his or her airway in the event of reflux or frank vomiting. Although none of the patients in our series reported having gastric reflux, a strong history of reflux may be an indication for rapid-sequence or awake intubation. When vocal cords and the immediate infraglottic areas are anesthetized, as is necessary for awake fiberoptic intubation, the cough reflex is suppressed, and it may become more difficult for patients to manage secretions. The use of rapid-sequence induction in patients who fasted with no risk factors for aspiration other than obesity is debatable.

Airway management in morbidly obese patients is extremely challenging and can result in serious adverse events and even death if mishandled. Poor satisfaction rates have been recorded despite fiberoptic bronchoscopy-guided awake intubation being the best choice for obese patients. The advantage of oxygenating morbidly obese patients with 100% oxygen cannot be overestimated, given the low functional residual capacity and rapid desaturation rates in this population. Thus, the ability to deliver high oxygen rates while using the CTrach™ to secure a view of the vocal cords is a significant feature of the SaCoVLM^TM^. Furthermore, it has been observed that ILMA is actually placed better in obese patients than in patients with a normal body habitus (8, 15).

Anatomical and /or physiological compromise can result in morbidity and mortality, which were common problems for Obesity and obstructive sleep apnea patients in the extubation time. Based on the management guideline for adult perioperative extubation issued by DAS in 2012 and the practical guide for difficult airway management issued by ASA in 2013 (1, 16), LMA as a replacement of tracheal intubation is superior to either awake or deep extubation (17, 18). However, it is inappropriate in patients in whom re-intubation would be difficult or if there is a risk of regurgitation. If SaCoVLM^TM^ can effectively ensure ventilation after implantation, we choose to remove tracheal intubation under deep anesthesia. Resuscitation with SaCoVLM^TM^ can be monitored visually while avoiding the risk of re-intubation. All 3 patients recovered well. This is the first time to report on this method. Of course, we will continue to study it in depth to support this view.

## Conclusion

In summary, this is the first reported series of awake insertions of the SaCoVLM^TM^ LMA in morbidly obese patients with difficult airways. Intubation with the SaCoVLM^TM^ LMA can provide oxygen for patients at any time. Its simple operation, well tolerated, and high success rate makes it a better airway management implementation tool in our practical work.

## Data Availability Statement

The original contributions presented in the study are included in the article/supplementary material, further inquiries can be directed to the corresponding author/s.

## Ethics Statement

The studies involving human participants were reviewed and approved by Ethics Committee of the First Affiliated Hospital of Shandong First Medical University. The patients/participants provided their written informed consent to participate in this study. Written informed consent was obtained from the individual(s) for the publication of any potentially identifiable images or data included in this article.

## Author Contributions

YS and YW conceived and wrote the manuscript. LH, LX, and YG collected patient's clinical information. YS and MZ analyzed and evaluated the treatment and curative effects. YW guided the entire process in terms of theory and practice and revised the manuscript. All other authors contributed to the analysis, reviewed results, and reviewed the manuscript.

## Funding

This work was supported by Shandong Provincial Health Commission, General Project, 2019-0388, Clinical study of oral ginkgo biloba extract EGB761 in the treatment of cognitive dysfunction after coronary artery bypass surgery, October 2019 to September 2022, 20,000.0 RMB. Jinan Science and Technology Bureau, Clinical Medicine Technology Innovation Plan, 202019170, Predictive study of peripheral blood biomarkers on cognitive dysfunction after cardiac surgery, January 2021 to December 2022, 100,000.0 RMB. Shandong First Medical University, Academic Improvement Program, 2019QL015, Perioperative lung protection and mechanical ventilation lung injury lung fibrosis gene regulation, July 2019 to June 2024, 20 million RMB.

## Conflict of Interest

The authors declare that the research was conducted in the absence of any commercial or financial relationships that could be construed as a potential conflict of interest.

## Publisher's Note

All claims expressed in this article are solely those of the authors and do not necessarily represent those of their affiliated organizations, or those of the publisher, the editors and the reviewers. Any product that may be evaluated in this article, or claim that may be made by its manufacturer, is not guaranteed or endorsed by the publisher.
